# Reporting guidelines for burden of disease studies: why and how?

**DOI:** 10.1093/eurpub/ckac130.097

**Published:** 2022-10-25

**Authors:** B Devleesschauwer, JA Haagsma, P Charalampous, R Assunção, C Di Bari, V Gorasso, I Grant, H Hilderink, J Idavain, T Lesnik, M Majdan, M Santric-Milicevic, E Pallari, SM Pires, D Plass, GMA Wyper, E Von der Lippe

**Affiliations:** European Burden of Disease Network, COST Action CA18218

## Abstract

**Background:**

The Disability Adjusted Life Year (DALY) is a frequently used metric to assess burden of disease (BoD). Many independent BoD studies have been performed across Europe, showing wide variations and inconsistencies in the application and reporting of DALY specific methods. The European Burden of Disease Network (burden-eu) aims to develop guidelines for reporting DALY calculation studies which may enhance transparency and comparability of BoD estimates across Europe and beyond.

**Methods:**

A burden-eu working group of experts generated a list of potential reporting items based on existing literature, guidance for developing guidelines and consultations with BoD experts. To pilot the drafted product, we asked BoD experts and non-experts to apply it to existing BoD studies. We received feedback and we revised the guidelines accordingly.

**Results:**

The guide for DALY calculation studies comprises about 25 items that should be reported in BoD studies. We included information about the study setting, data input sources including methods for data corrections, DALY-specific methods (e.g., YLL life table, YLD approach, disability weights etc), data analyses, and data limitations. We also included information on how users can compare their new estimates with previously available BoD estimates.

**Conclusions:**

We introduced a reporting instrument for DALY calculations that can be used to document input data and methodological design choices in BoD studies. The application of such guidelines will enhance usability of BoD estimates for decision-makers as well as global, regional, and national health experts.

**Key messages:**

- Application of reporting guidelines will increase consistency and transparency in reporting of BoD studies, thus enhancing usability of BoD estimates.
- Reporting guidelines for BoD studies will serve as an educational tool for better understanding the complexity of DALY methodological design approaches.

